# Identification of selective cytotoxic and synthetic lethal drug responses in triple negative breast cancer cells

**DOI:** 10.1186/s12943-016-0517-3

**Published:** 2016-05-10

**Authors:** Prson Gautam, Leena Karhinen, Agnieszka Szwajda, Sawan Kumar Jha, Bhagwan Yadav, Tero Aittokallio, Krister Wennerberg

**Affiliations:** Institute for Molecular Medicine Finland, University of Helsinki, Helsinki, Finland

**Keywords:** TNBC, Chemosensitivity, Viability, Cytotoxicity, DSRT, DSS, Heterogeneity, Synergy, Antagonism

## Abstract

**Background:**

Triple negative breast cancer (TNBC) is a highly heterogeneous and aggressive type of cancer that lacks effective targeted therapy. Despite detailed molecular profiling, no targeted therapy has been established. Hence, with the aim of gaining deeper understanding of the functional differences of TNBC subtypes and how that may relate to potential novel therapeutic strategies, we studied comprehensive anticancer-agent responses among a panel of TNBC cell lines.

**Method:**

The responses of 301 approved and investigational oncology compounds were measured in 16 TNBC cell lines applying a functional profiling approach. To go beyond the standard drug viability effect profiling, which has been used in most chemosensitivity studies, we utilized a multiplexed readout for both cell viability and cytotoxicity, allowing us to differentiate between cytostatic and cytotoxic responses.

**Results:**

Our approach revealed that most single-agent anti-cancer compounds that showed activity for the viability readout had no or little cytotoxic effects. Major compound classes that exhibited this type of response included anti-mitotics, mTOR, CDK, and metabolic inhibitors, as well as many agents selectively inhibiting oncogene-activated pathways. However, within the broad viability-acting classes of compounds, there were often subsets of cell lines that responded by cell death, suggesting that these cells are particularly vulnerable to the tested substance. In those cases we could identify differential levels of protein markers associated with cytotoxic responses. For example, PAI-1, MAPK phosphatase and Notch-3 levels associated with cytotoxic responses to mitotic and proteasome inhibitors, suggesting that these might serve as markers of response also in clinical settings. Furthermore, the cytotoxicity readout highlighted selective synergistic and synthetic lethal drug combinations that were missed by the cell viability readouts. For instance, the MEK inhibitor trametinib synergized with PARP inhibitors. Similarly, combination of two non-cytotoxic compounds, the rapamycin analog everolimus and an ATP-competitive mTOR inhibitor dactolisib, showed synthetic lethality in several mTOR-addicted cell lines.

**Conclusions:**

Taken together, by studying the combination of cytotoxic and cytostatic drug responses, we identified a deeper spectrum of cellular responses both to single agents and combinations that may be highly relevant for identifying precision medicine approaches in TNBC as well as in other types of cancers.

**Electronic supplementary material:**

The online version of this article (doi:10.1186/s12943-016-0517-3) contains supplementary material, which is available to authorized users.

## Background

The triple negative subtype of breast cancer (TNBC), devoid of the hormone estrogen/progesterone receptor (ER/PR+) expression and HER2 overexpression (HER2+), is a heterogeneous group of aggressive diseases that account for 15–20 % of all breast cancer cases. While targeted treatments exist for the receptor positive breast cancer subtypes, TNBC lacks such specific treatments. The current line of therapy is limited to surgery, radiation and chemotherapy [1, 2]. TNBC patients have a worse prognosis than other breast cancer patients. In a breast cancer patient follow-up study, 93 % 5-year survival was seen in non-TNBC-patients, as compared to only 77 % of the TNBC-patients [3]. Hence, there is an obvious need for better treatment options for TNBC.

Development of targeted therapeutics for TNBC diseases is challenging due to their heterogeneity. To address this challenge, several studies have assigned TNBC cases into multiple subtypes using transcriptomics approaches. For example, Kreike et al. [1] studied 97 TNBC samples and 7700 genes and observed five groups (I-V) in a hierarchical analysis of gene expression data. In another study, TNBC was grouped into seven transcriptomics-based subtypes: basal-like 1 (BL1) and 2 (BL2), mesenchymal-like (M), mesenchymal stem cell-like (MSL), immunomodulatory (IM), luminal androgen receptor positive (LAR) type, and unclassified (UNC) [4]. Recently, Burstein et al. [5] performed a similar study and identified four subtypes; LAR, MES, BLIS and BLIA. By assessing expression of 13 biomarkers, TNBC was assigned to four subtypes by Elsawaf et al. [6]. Based on intrinsic PAM50 subtyping, 80.6 % of TNBC were found basal-like, 14.6 % normal-like, 3.5 % luminal A, 1.1 % luminal B and 0.2 % HER2-enriched [7, 8]. While there is overlap between the results in the different studies, such as identification of the LAR-type in two recent studies [1, 5], it is evident that defining clear, distinct subgroups is challenging, highlighting the diversity of the disease.

Despite the diversity, a number of therapy-guiding biomarkers have been proposed and efforts for tailoring targeted therapies against TNBC are ongoing. Though *TP53*, *BRCA1/2*, *EGFR*, *PIK3CA* and *PTEN* tend to be dominant mutations in TNBC, these markers have been elusive and inconsistently useful for guiding therapy [9, 10]. An important finding is that Poly-ADP-ribose polymerase (PARP) inhibitors appear to be highly effective against the *BRCA1*-mutant TNBC [11]. The PARP inhibitor olaparib was recently approved for use in *BRCA*-mutated ovarian cancers and several PARP inhibitors are currently in clinical trials against *BRCA*-mutated TNBC. Furthermore, inhibitors of PI3K, mTOR, CDK, HDAC and androgen signaling are currently being explored in clinical trials as treatments of TNBC [8]. Also drugs targeting different growth factor receptors such as EGFR, VEGFR and FGFR are explored in clinical trials [7].

As an alternative strategy to tailor targeted therapies to breast cancers, chemosensitivity profiling of in vitro cell lines is applied increasingly. This functional profiling approach allows for identification of selective vulnerabilities in cell lines reflecting human diseases. Recently, Barretina et al. [12] and Garnett et al. [13] tested 25 TNBC lines against 24 anticancer agents and 10 TNBC lines against 130 compounds, respectively, as part of large comprehensive pharmacogenomics studies in hundreds of cell lines. Heiser et al. [14] performed an analysis of 77 cancer drug compounds on 19 TNBC cell lines, and combined the drug-sensitivity data with gene expression and copy number interrogation. In a similar study by Daemen et al. [15], 19 TNBC cell lines were screened against 90 compounds along with integration of multi-omic molecular profiling data to identify potential response-predictive markers. Another study by Lawrence et al. [16] reported a combined proteomics, genomics, and drug sensitivity interrogation using 160 compounds and 16 TNBC cell lines and four tumor samples. Muellner et al. [17] identified the broad-spectrum tyrosine kinase inhibitor midostaurin (PKC412) as a post-EMT-specific drug targeting spleen tyrosine kinase SYK in a subset of TNBC cells.

Together, these studies identified a number putative links between drug sensitivities, TNBC subtypes and genomic and proteomic markers, but also highlighted a striking functional heterogeneity among TNBC cell lines. Notably, each of these studies used cell viability readouts to monitor the drug responses. However, the consistency in Barretina et al. and Garnett et al. datasets [18] was poor, possibly due to differences in experimental setup and especially in their viability readouts. These results highlight the importance of the well-defined functional readouts in chemo-sensitivity profiling studies.

By studying the response of 16 diverse TNBC cell lines to 301 compounds using our drug sensitivity and resistance (DSRT) approach [19, 20], we investigated whether going deeper than the traditional cell viability measurement could provide us with improved drug response selectivity information of translational value. Our compound testing included a resazurin cytosolic reduction-based viability assay, a cellular ATP-based viability assay, and a cell membrane impermeable DNA-binding dye-based cytotoxicity assay [21]. While the readouts of the two viability assays correlated well, we found that several classes of drugs caused an apparent drastic loss in viability and cell numbers but failed to induce cell death. However, among these generally cytostatic agents, sporadic cytotoxicity occurred in specific cell lines, suggesting that some cell lines, and presumably cancers with the same genotype and/or phenotype, may be strongly and selectively responsive to such chemotherapy. Furthermore, when testing drug combinations, the cytotoxicity readout revealed both antagonistic and synthetic lethal effects that were missed by the viability readouts.

## Results

### Viability and cytotoxicity readouts reveal differential drug responses

**Fig. 1:**
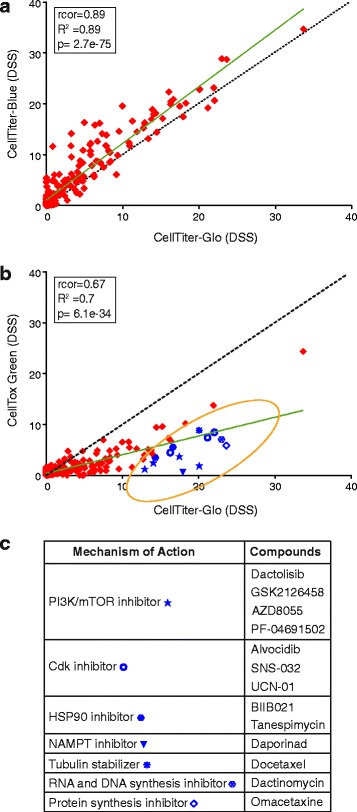
A number of viability-inhibiting compounds do not induce cell death. Scatter plot showing the comparison of average DSS over the panel of 19 different breast cancer cell lines computed using two **a** independent viability measurement assays (CellTiter-Blue and CellTiter-Glo) and **b** viability measurement (CellTiter-Glo) and cell death measurement (CellTox Green) assays. The black dotted line represents the equal score in the screens and the green solid line represents the linear regression. Blue symbols mark compounds with strong viability readout responses but with low to no cytotoxicity. **c** The major classes of compounds highlighted in panel B that inhibit the viability readout of the cells but do not effectively induce cell death

Most drug sensitivity screening studies have been performed using cell viability assays. As such assays are based on changes in cellular metabolism, we first set out to investigate whether a viability assay was sufficient to reveal cell death. A panel of 19 breast cancer cell lines (Additional file 1: Table S1) was screened against 301 oncology substances (Additional file 2: Table S2) (DSRT, [19]) using two cell viability detection reagents, the resazurin cytosolic reduction-based detection reagent CellTiter-Blue and the luciferase-based cellular ATP detection reagent CellTiter-Glo; and a cell death detection reagent, the cell impermeable DNA-binding dye CellTox Green. The drug sensitivity score (DSS), a measure of drug response based on the area under the dose response curve that therefore captures both the potency and the efficacy of the drug effect, was calculated for each compound, as previously described [19, 20], and an average response for each compound was summarized (Additional file 3: Table S3). The DSS responses from the two independent viability measurement assays (CellTiter-Blue vs. CellTiter-Glo) were highly correlated (*R*^2^ = 0.89; Fig. 1a). In contrast, a more heterogeneous response was observed between the cell viability (CellTiter-Glo) and cytotoxicity (CellTox Green) assays (*R*^2^ = 0.67; Fig. 1b). Overall, the compounds affected the cellular ATP-levels considerably more than cell death. A large group of compounds appeared to inhibit the cell viability, but the same compounds failed to induce cell death to a similar extent. Compounds that displayed such effect across the cell panel included PI3K/mTOR inhibitors, Cyclin-dependent kinase (CDK) inhibitors, Heat shock protein 90 (HSP90) inhibitors, an NAMPT inhibitor, tubulin stabilizing anti-mitotics; and protein, RNA and DNA synthesis inhibitors (Fig. 1c). These results suggest that inhibition of signal in cell viability assays is not directly indicative of cell death.

### Drug response patterns do not link to transcriptomics-based grouping of TNBC

**Fig. 2:**
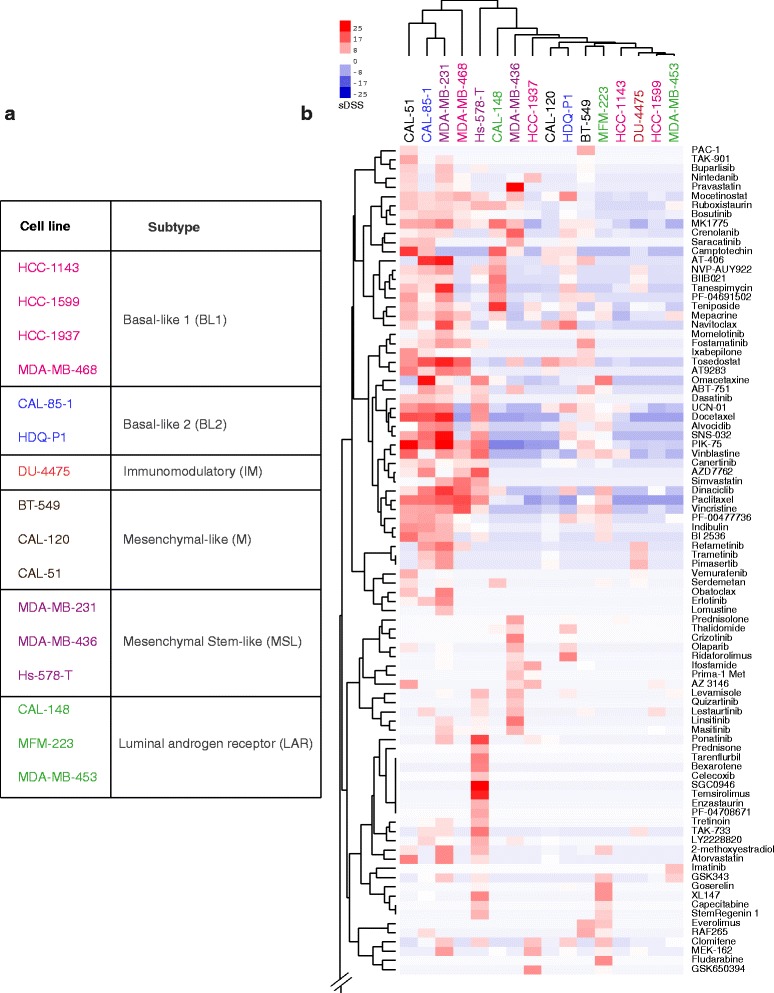
Drug sensitivity driven clustering is not linked to transcriptomics subtyping of TNBC cell lines. **a** Table representing the classification of TNBC cell lines according to gene expression profile according to Lehmann et al. [4]. The cell lines are assigned with different colors to represent different subtypes. **b** Heat map based on selective DSS (toxicity, as compared to a control set of 30 cell lines) with compound clustering shown vertically and cell line clustering horizontally. Only a portion of the compound set is shown. See Additional file 5: Figure S1 for full heat map. The cell line clustering based on drug vulnerabilities fails to resemble transcriptomics-based classifications (subtypes color coded as per table on the left)

Next we investigated whether the heterogeneous responses of different TNBC cell lines to the 301 oncology compounds could be linked to the previously published, gene expression profile-based TNBC subtypes: basal-like 1 and 2 (BL1, BL2), immunomodulatory (IM), mesenchymal like (M), mesenchymal stem cell-like (MSL) and luminal androgen receptor expressing (LAR) [4]. The panel of TNBC cell lines represented the six subtypes (Fig. 2), and for control purposes, two HER2 receptor positive (SK-BR-3, BT-474) cell lines, and a non-cancerous triple negative breast epithelial cell line MCF-10A were added. A selective DSS (sDSS) against the average DSS for each compound was calculated from in-house screening data of a large set of cell lines originating from different tissue types (Additional file 4: Table S4).

None of the compounds exhibited selectivity against all TNBC cell lines screened. Instead, the cell lines exhibited highly diverse responses towards the compound panel. When comparing the DSRT data to the published gene expression-based TNBC subgrouping [4], DSRT-driven clustering was not linked to the transcriptomic subtyping of the cell lines (Fig. 2, Additional file 5: Figure S1 & Additional file 6: Figure S2). The cytotoxicity-based drug-response clustering analysis divided the cell lines into two groups; the toxicity sensitive and insensitive groups. Five cell lines; CAL-51, CAL-85-1, MDA-MB-231, MDA-MB-468 and Hs-578-T stood out from the group as they showed higher vulnerability (cell death) towards several types of compounds such as *Vinca* alkaloids, mitotic-, CDK-, topoisomerase- and HDAC- inhibitors along with various discrete sensitive responses towards other kinase inhibitors and other small molecules (Fig. 2). These results argue that personalized therapeutic strategies based on functional profiling can be a more effective way to target TNBCs rather than therapies based on transcriptomics subtyping.

### Non-toxic cell viability responses represent a reversible cell growth arrest

**Fig. 3:**
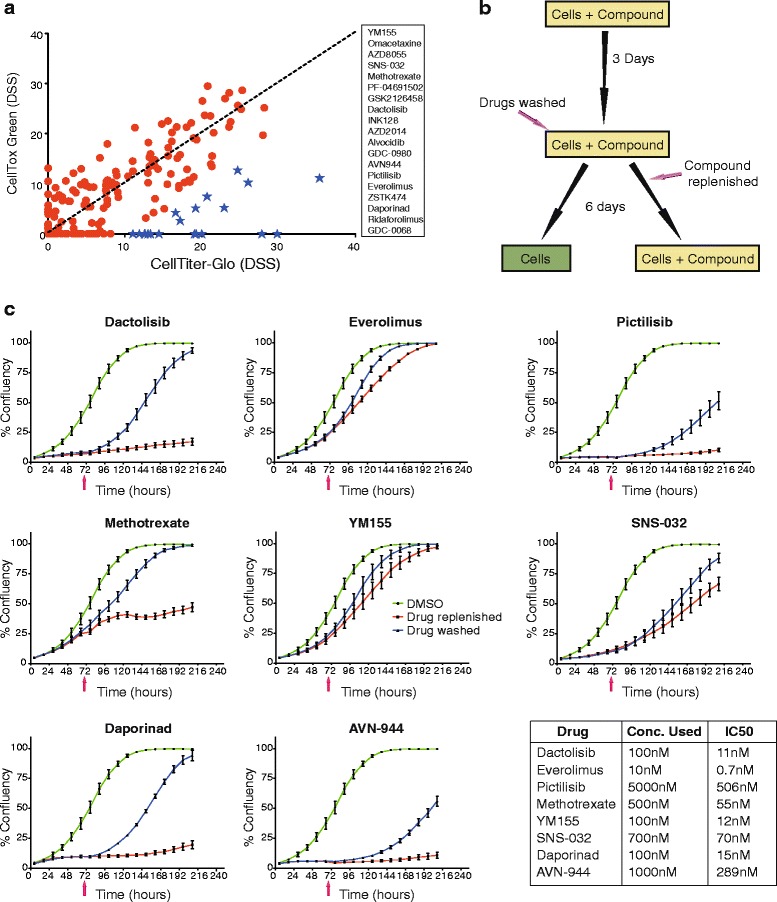
mTOR inhibitors and mitotic inhibitors cause cytostatic but not cytotoxic effects in CAL-51. **a** Scatter plot comparing DSS for CAL-51 computed using viability assay (CellTiterGlo) and cell death assay (CellTox Green). Some compounds caused both viability inhibition and cytotoxicity, but a large number of compounds (represented with blue stars and listed on the right-hand side of the plot) showed high degree of viability inhibition with little or no induction of cell death. **b** Schematic illustration of experimental workflow. **c** Growth curves affected by selected highlighted drugs in plot (**a**) showing their effect in viability inhibition is due to arrest in cell cycle rather than induction of cell death. CAL-51 cells were cultured in 96-well plates with compounds for 72 h at which point the inhibitors were either washed away or replenished (time indicated with pink arrow). Growth measured as confluency was monitored and calculated using an IncuCyte Zoom live cell microscope for 9 days. Cell growth was arrested in the presence of methotrexate, dactolisib, daporinad, AVN-944 and pictilisib; and released upon removal of the compounds. Similarly, everolimus, SNS-032 and YM155 initially arrested cell growth but eventually growth was restored, also in the presence of the compounds, pointing to a rapidly established adaptive resistance

As a number of compounds caused dramatic changes in cell viability but failed to kill the cells, we next explored whether this reflected a reversible or non-reversible response. Eight different compounds that showed strong viability inhibition but were non-toxic against most of the tested cell lines were selected: dactolisib (targeting mTORC1 and mTORC2), everolimus (mTORC1), pictilisib (PI3Ks), methotrexate (folate metabolism), YM155 (survivin), SNS-032 (CDK2, 7 & 9), daporinad (NAMPT) and AVN-944 (IMPDH) (Fig. 3a). To explore the mechanism of the observed non-toxic cytostasis, CAL-51 was selected as the model cell line.

Using a drug effect reversibility test in which compounds were removed after 72 h followed by several days further incubation (Fig. 3b), the static effects of the 8 compounds were all found to be reversible. In some cases, the inhibitory effect of the drug was overcome even in the presence of the drug during the 9-day experiment. In the presence of dactolisib, pictilisib, daporinad and AVN-944, the cell growth was arrested or strongly inhibited; yet the cells began dividing again when the compounds were washed away (Fig. 3c). Methotrexate, everolimus, YM155 and SNS-032, on the other hand, only caused a transient inhibitory effect that was lost within two to five days, as the cells began to grow even in the presence of the compounds (Fig. 3c). Hence, the non-toxic cell viability responses are cytostatic and reversible, sometimes even in the presence of the inhibitor.

### Broad-acting cytostatic compounds exhibit selective toxic responses that can be linked to protein expression signatures

**Fig. 4:**
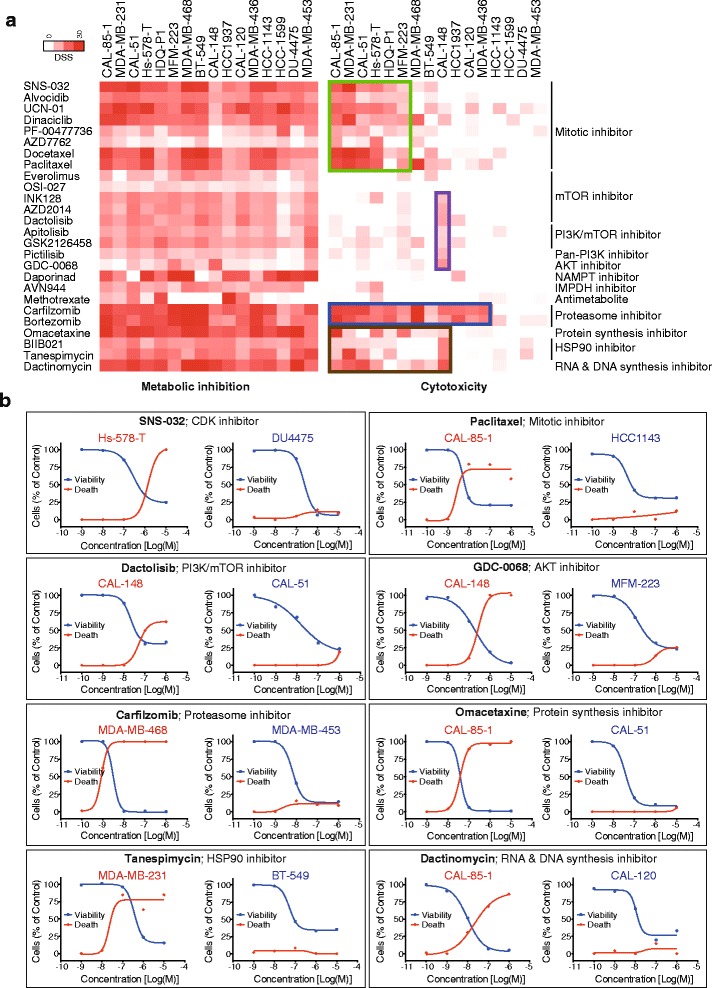
Viability inhibition does not correlate with cell death. **a** Comparison of DSS heat map, cell viability (CellTiter-Glo) vs. cell death (CellTox Green) assays. Cell death was induced only in specific cell lines. Specific cell death is highlighted for different classes of drugs using colored boxes. **b** Examples of selective cytotoxic responses among different cell lines. Each box represents specific effect of different drug classes. mTOR and AKT inhibitors specifically induced cell death only in CAL-148

Next, we focused on the compounds that exhibited broad cytostatic effects on all or most of the cell lines. Among these agents, highly heterogeneous effects on cytotoxicity were discovered in the TNBC cell lines (Fig. 4a). PI3K-AKT-mTOR pathway inhibitors were almost exclusively cytostatic across the panel with CAL-148 being the only cell line showing a cytotoxic response. The metabolic inhibitors methotrexate, daporinad and AVN944 were unable to induce cell death in most of the cell lines in contrast to their strong responses in viability readouts. CDK-inhibitors and conventional antimitotics induced cytotoxicity in six TNBC cell lines while the other cell lines were unresponsive.

**Fig. 5:**
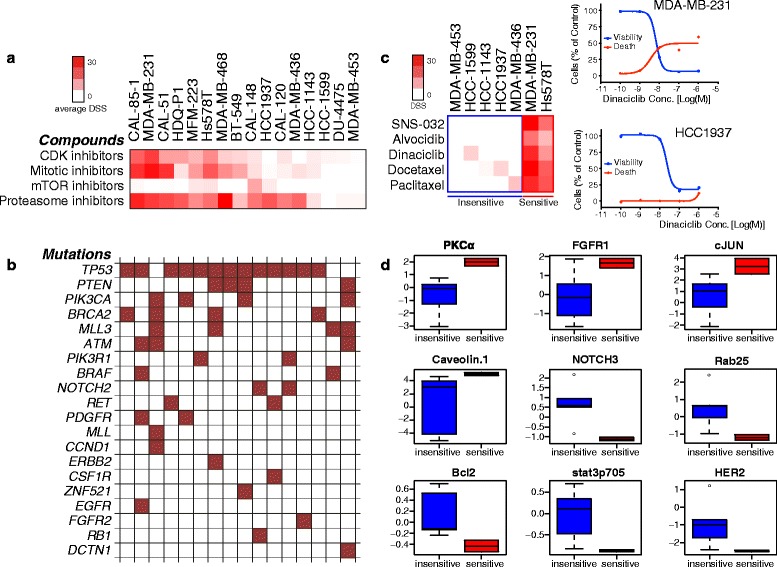
Potential predictive biomarkers for cytotoxic effect of mitotic drugs. **a** Evaluation of recurrent genetic alteration in the studied 16 TNBC cell lines. The heat map in panel **a** represents the average cytotoxic response of CDK-, mitotic-, mTOR- and proteasome inhibitors whereas in panel **b** heat map represents recurrent mutations in each of the cell lines. The analysis failed to link the cytotoxic responses to any mutation detected. **c** Heat map highlighting/differentiating mitotic inhibitor-sensitive and insensitive cell lines based on cytotoxicity exhibited by 5 different mitotic drugs. The dose response curves (right) illustrate the effects of the representative mitotic inhibitor dinaciclib on cell viability and toxicity in two individual cell lines. **d** Box plots showing the differential expression of protein and phosphoprotein levels (from Daemen et al. [15]) between mitotic inhibitor sensitive and insensitive cell lines

Proteasome inhibitors induced potent cell viability responses in nearly all but failed to cause toxicity in four cell lines. Similarly, the HSP90 inhibitors BIIB021 and tanespimycin induced death in seven cell lines whereas the nucleic acid synthesis inhibitor dactinomycin induced death in nine cell lines. To explore whether these heterogeneous toxic responses could be predicted from molecular markers, we investigated links between known recurring genetic alterations in the cell lines and took advantage of the protein marker expression profiles published by Daemen et al. [15] in which 8 out of our 16 TNBC cell lines were included. Selective toxic responses could not be linked to particular gene mutations (Fig. 5b). On the other hand, in comparing the Daemen protein expression data set with our toxicity response profiles, cell lines exhibiting cytotoxic responses to taxane antimitotics could be linked to high expression levels of a set of proteins, including PKCα, FGFR1, c-Jun, Caveolin-1 and low expression levels of NOTCH3, RAB25, Bcl-2, STAT3(pY705) and HER2 as compared to the taxane-insensitive cell lines (Fig. 5d). Similarly, stratified toxic responses to proteasome inhibitors linked to high levels of PAI1, MPK-1, AKT(pT308), p38(pT180/Y182) and low levels of NOTCH3, CCND1 and PTEN (Additional file 7: Figure S3). Thus, the heterogeneous cytostatic effect of mitotic and proteasome inhibitors may be linked to differentially expressed protein markers.

### mTOR inhibitors antagonize the effects of diverse classes of drugs

**Fig. 6:**
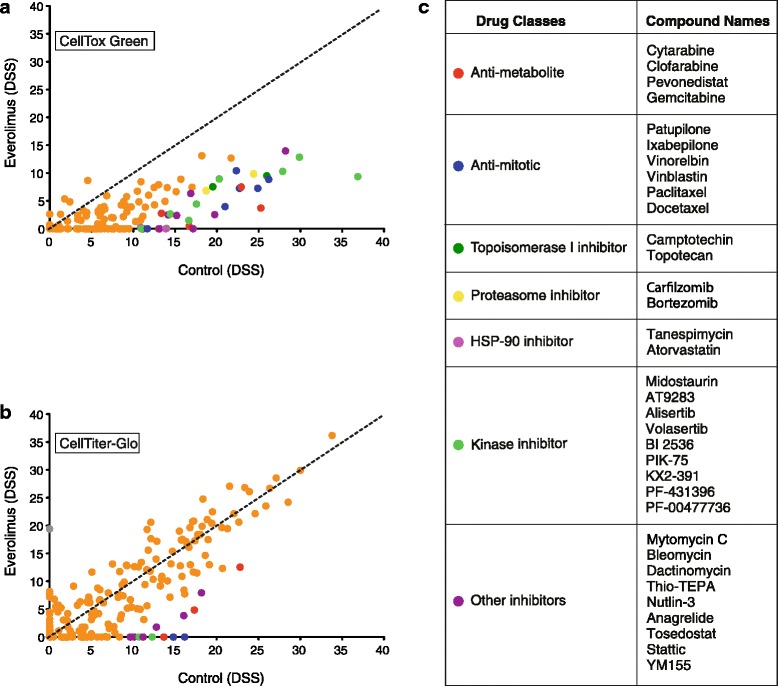
An mTOR inhibitor antagonizes the effect of diverse classes of drugs in CAL-51. Scatter plot of DSS scored for combinatory effect of different compounds along with everolimus (10 nM) (Y-axis) on CAL-51 cell line compared to the single agent effects (X-axis). Plot **a** represents the DSS computed using cell death assay and plot **b** represents cell viability assay. Data points with DSS difference more than 10 are highlighted with different colors representing different classes of drugs as listed in the color legend. **c** Compounds whose effect is inhibited when combined with everolimus, as highlighted in panels (**a**) and (**b**)

As the cellular responses to mTOR inhibitors were almost exclusively non-cytotoxic, we tested whether combining mTOR inhibitors with other compounds would improve their cytotoxicity. To this end, single concentrations of mTOR inhibitors everolimus (10 nM) or dactolisib (100 nM) were combined with the full oncology compound library and responses of CAL-51, an mTOR inhibitor-sensitive cell line based on viability data, were tested (Fig. 6 and Additional file 8: Figure S4, respectively). Instead of identifying synergistic compounds, the mTOR inhibitors were found to have unanticipated antagonistic effects on the activity of other cancer compounds in both viability and toxicity readouts, including conventional chemotherapeutics: anti-metabolites, *vinca* alkaloids, taxanes, antitumor antibiotics and proteasome inhibitors. Daporinad, an NAMPT inhibitor, appeared to be synergistic in combination with everolimus, but the combination was not cytotoxic. In summary, mTOR inhibitors not only fail to kill TNBC cells but can also antagonize the cytotoxicity of most other anti-cancer compounds.

### Combination studies show selective synergistic cytotoxic effects not seen in viability measurement

**Fig. 7:**
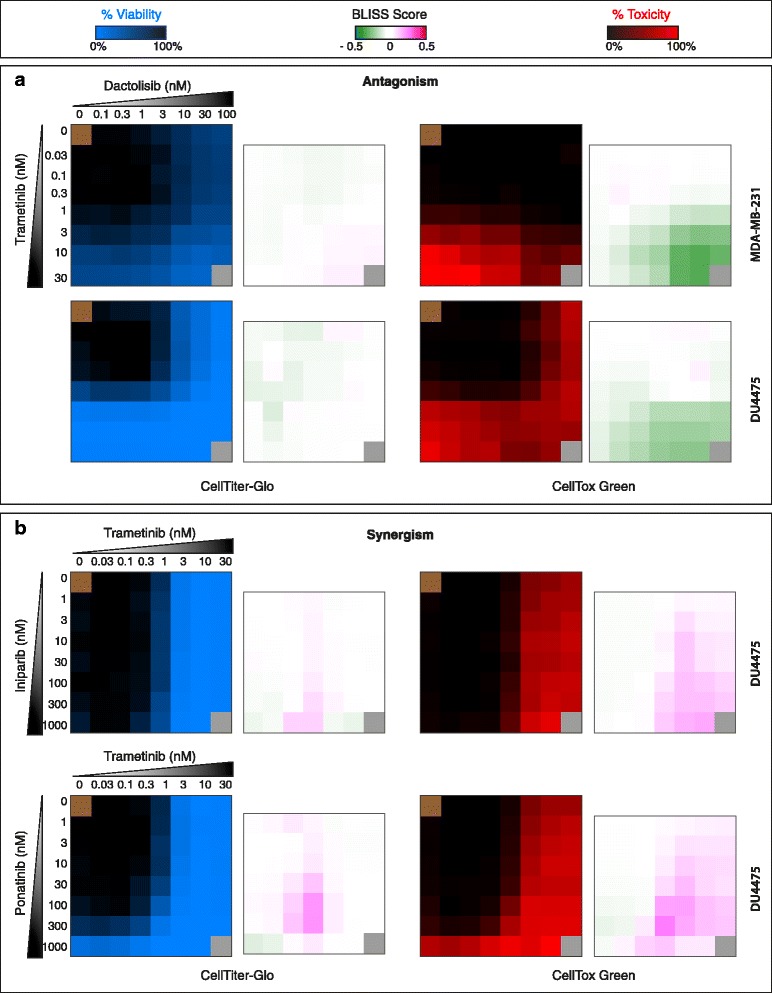
Assessing both cell viability and cell death is necessary to evaluate drug combination effect. Seven different concentrations of two different drugs were combined in 8 × 8 matrix format, in which blue matrix shows % inhibition in viability whereas red matrix shows % cell death (toxicity), and adjacent 7 × 7 matrix represents the combined effects of the drugs (Bliss synergy score), in which pink indicates synergy and green antagonism. **a** In MDA-MB-231 and DU4475 combination of dactolisib and trametinib showed additive effect in enhancing viability inactivation whereas dactolisib antagonized the cytotoxicity of trametinib in both cell lines. **b** In DU4475 combination of trametinib with iniparib and ponatinib enhanced both viability inactivation and cell death effect but it is more pronounced in cytotoxicity readout than in the viability readout

On combining a single concentration of MEK inhibitor and mTOR inhibitors to our oncology compound collection, we observed several interesting combination effects in both viability and cytotoxicity measurements. Hence, we compiled a number of combinations based on our screening to be tested in drug concentration combination matrices. The concentration combination matrices were set up in which seven different concentrations of two drugs were combined in an 8 × 8 matrix. Combining the MEK inhibitor trametinib either with the PARP-inhibitors iniparib and olaparib, or with the broad-spectrum tyrosine kinase inhibitor ponatinib showed synergistic cytotoxic combination responses in DU4475 cells (but not in CAL-148, Additional file 9: Figure S5B). Combining the mTOR inhibitor dactolisib with trametinib resulted in additive viability inhibition but striking antagonistic cytotoxicity responses (Fig. 7a); whereas targeting mTOR using both a rapamycin analog and an ATP competitive inhibitor surprisingly resulted in a synergistic viability inhibition and synthetic lethal cytotoxicity to cell lines DU4475 and CAL-148 (Additional file 9: Figure S5A). The cytotoxicity readout allowed us to identify effective synergistic drug combination concentrations that were not revealed using the cell viability readouts.

Using viability readouts, higher concentrations of trametinib alone saturated the effect (Fig. 7b). Additional file 10: Table S5 represents the data for Fig. 7 and Additional file 9: Figure S5. In conclusion, studying drug-induced cytotoxicity is a potent way of identifying effective individualized drug combinations using in vitro and ex vivo drug sensitivity testing.

## Discussion

In this study, we systematically explored how comprehensive drug responses with different cell health readouts compared to current subgroupings and previously described biomarker information of triple-negative breast cancers. The results led us to several conclusions. First, the drug response clustering of the TNBC cell lines based on their differential drug vulnerabilities resulted in highly heterogeneous patterns of drug responses and distinctive grouping compared to gene expression derived grouping. Second, by studying cell death rather than cell viability, which has so far been the standard readout in other large-scale chemosensitivity profiling studies, we could separate static responses from the cytotoxic ones and identify several drug classes that exhibit broad viability readout effects but induce only limited or no cell killing responses. Third, the cytostatic responses seen by many drugs were reversible, and by studying the cells in real-time, we detected that some of the static responses were overcome even in the presence of the drugs. Fourth, by measuring the cytotoxic responses in drug combination studies, synergistic cytotoxic responses and even synthetic lethalities were detected that were not observed with the cell viability readout. In our combination studies, we explored the simultaneous exposure of compounds because we lacked the scientific evidence suggesting that one agent should be added before the other, and without that information, exploratory testing of many combinations in different addition orders became unmanageable in size and cost. However, as a proof of approach, we did perform combinatorial order of addition testing of dactolisib and trametinib in DU4475 and MDA-MB-231 to see if the antagonistic effects of this combination could be reverted by an appropriate sequential addition of the compound. In these experiments, the combination remained antagonistic in both cell lines regardless of whether simultaneous or sequential addition was applied (Additional file 11: Figure S8).

Similar to what others have shown [15, 22] we detected a great heterogeneity in drug responses among the TNBC cell lines, re-emphasizing the heterogeneous nature of this breast cancer subtype. As has also been shown by others as well as by us in other cell systems [19], the overall drug response profiles are not easily linked to genetic or transcriptional profiles, arguing that functional drug response profiling is currently the most powerful way to identify individualized vulnerabilities that can be used to target the disease. Alternatively, a more refined analysis of TNBC transcriptomics may be needed for effective linking to broad drug sensitivities.

In vitro anti-cancer chemosensitivity testing has traditionally been focused on growth inhibition measurements with the assumption that reducing or stopping cancer cell growth will translate into an anti-cancer activity of the agent in vivo. We hypothesized that by also following the drug-induced cytotoxicity, one can discover a deeper and different range of drug responses, which may also lead to more translationally-predictive results. Overall, our results with the cytotoxicity measurement strongly argue that high throughput chemosensitivity profiling of cancer cells need to go beyond the current standard viability measurement. There are various types of viability measurement reagents commonly employed in multiwell-based assays but most of them monitor the metabolic activity of the cells, measuring the amount of energy molecules like ATP, NADH, NADPH or the redox activity in the cells. Here, we show that using two different commonly used viability readouts, one measuring cellular ATP and one measuring reducing potential of the cells give highly correlated results. In other chemosensitivity profiling studies, it has often been implied that the loss in apparent cell viability should also strongly correlate to cytotoxicity. However, our data clearly show that this is often not the case. Only some of the compounds that appeared effective when assessed using cell viability readouts, were able to potently induce cell death. The easily adaptable multiplexed cell viability and cell death readout we applied allowed us to identify several drugs and drug classes that inhibited viability across the 19 breast cancer cell lines but failed to induce broad cell death responses. These included PI3K/mTOR inhibitors, CDK inhibitors, HSP90 inhibitors, anti-metabolites and antimitotic drugs. Importantly, we also showed that these cytostatic responses were fully reversible. Cells started growing as soon as inhibitory compounds were removed. Furthermore, in some cases (such as with rapamycin analogs and some CDK inhibitors), cell growth inhibition was bypassed over time even in the presence of the compounds, presumably an effect of cellular reprogramming in response to the drug as has been described in other model systems [23, 24]. Among the compounds that caused a very preferential cytostatic effect, there was still heterogeneity in cytotoxic responses within the cell line panel. In most cases, there were subsets of the cell lines that exhibited strong cytotoxic responses, such as CAL-85-1, MDA-MB-231, CAL-51, Hs-578-T, to antimitotic taxanes and we hypothesize that these selective cytotoxic responding cell lines represent the TNBC subgroups that are more likely to respond to each specific type of therapy.

PI3K/AKT/mTOR signals have gained attention as potential therapeutic targets for several cancer types [25–27]. Our results suggest that PI3K/mTOR inhibitors are able to induce viability inhibition in most of the cells lines, but fail to induce selective cell death. Given that mTOR inhibition is expected to slow down or halt cellular metabolism and thereby cell growth, our finding is not surprising *per se*, but it emphasizes that using a cell viability/metabolic readout most likely is not relevant when assessing the effects of PI3K, AKT and mTOR inhibitors in vitro or ex vivo. Judging from the cell death readout, all the cell lines except CAL-148 were unresponsive or only had weak responses towards the inhibitors that target the PI3K/AKT/mTOR pathway. Furthermore, mTOR inhibitors, presumably through their antimetabolic activity, antagonize the activity of diverse classes of compounds, including many conventional antimitotic and cytotoxic drugs. Numerous clinical trials for PI3K and mTOR inhibitors along with conventional chemotherapy are ongoing, but our results argue that combining mTOR inhibitors with traditional chemotherapy such as doxorubicin, etoposide, gemcitabine should be considered with caution as combinations might turn out to be counterproductive. However, as our study was carried out using cell cultures, it may not fully reflect the responses in the considerably more complex biological settings when treating a cancer patient.

We found that mitotic and proteasome inhibitors had a heterogeneous cytotoxic effect on TNBC cell lines. This led us to try to find biomarkers that could be linked to the cytotoxic effects of the mitotic and proteasome inhibitors. We compared the basal protein and phosphoprotein levels in cell lines that were either sensitive or insensitive to the mitotic and proteasome inhibitors. Despite of the small overlap between our cell line collection and Daemen et al. [15] study, we were able to identify some candidates that could potentially be further explored for predictive biomarkers.

The mitotic inhibitor-responsive cell lines expressed a higher level of the survival regulator PKCα [28]; of FGFR1 that has been linked to TNBC cell growth [25, 26]; of the cell cycle and apoptosis regulator c-Jun [27]; and of caveolin-1, low level of which has been linked to poor clinical outcome in TNBC [28]. The mitotic inhibitor-sensitive cell lines also expressed low levels of NOTCH3, which has been linked to induction of apoptosis in HER2-negative breast cancer cell lines [29, 30]; of the small GTPase protein Rab25, which has been linked to aggressiveness of epithelial cancers [31]; of Bcl-2 and Stat3 high expression of which have been linked to the development of chemoresistance [32–34] and of the well-known driver of chemoresistance, HER2 [35, 36].

Proteasome inhibitors have been found efficient against hematologic malignancies but less successful against solid tumors [37]. We discovered that the proteasome inhibitor-sensitive cell lines exhibited high level of PAI1, a well established prognostic biomarker for the selection of chemotherapy [38]; MKP-1 [39]; AKT and p38. Low levels of NOTCH3; the cell cycle regulator Cyclin D1 that has been linked to chemoresistance in multiple cancers [40, 41] and PTEN were observed in proteasome inhibitor-sensitive cell lines. Proteasome inhibition has been shown to activate phosphorylation of p38, MKP-1, and AKT that further activate resistance to proteasome inhibitors [42, 43]. Similarly, suppression of PTEN has been linked to chemoresistance [44]. Our findings suggest that some subgroups TNBC might be responsive to treatment with proteasome inhibitors.

In vitro/ex vivo drug sensitivity testing is a re-emerging area of research, thanks to improved possibilities to follow phenotypic drug responses and the possibility to link the responses to deep molecular profiling. However, there are limitations to such high throughput testing. Due to experimental logistics and scale it is still challenging to comprehensively address the complex pharmacology and metabolism of the compounds in vivo, extended time dependent effects of the drugs, as well as order of addition combination testing. Therefore, false negative results are always possible in these types of screening approaches. For example, some of the compounds in our collection represented prodrugs that are metabolized into active substances in the liver in vivo and are largely inactive in vitro. This group of compounds included most alkylating agents but these were still included in our collection because they are approved for human use. We also also attempted to use the active metabolites of several of these compounds in our screens but they were too unstable to be suitable for screening use. On the other hand, some other prodrugs, such as nucleoside analogs, are metabolized in the target cells and were therefore highly relevant to include in the in vitro testing. Furthermore, compounds with diverse mechanisms of action are likely to reach their cellular effects at different time points, a challenge when performing high throughput testing of broad arrays of agents where a single endpoint measurement becomes the most feasible assay readout. In our testing, we chose the 72 h endpoint for the experiments, as we found it sufficient to observe the activity of the majority of the compounds in our collection. Extending the incubation to up to 168 h did not significantly affect the overall results (Additional file 12: Figure S6), and hence the shorter time point was preferred because of assay logistics and robustness.

Here we studied cell lines, but novel technologies have emerged in recent years that may allow for systematically taking this approach on primary patient cells ex vivo, ultimately making it more directly translational. One example is the culture of 3-dimensional organoids closely recapitulating disease conditions [45]. Organoid culture methods have already been established for human mammary tissue and primary breast cancer cells [46, 47]. In an alternative approach, primary cells can be cultured on fibroblast feeder cells in the presence of a Rho kinase inhibitor resulting in immortalized, conditionally reprogrammed progenitor-like cells. This approach allows for 2- or 3-dimensional culture of patient-derived cells that maintain the heterogeneity of the initial tissue environment [48, 49]. Finally, the prospect of generating these types of cultures from either circulating tumor cells or biopsies opens the possibility to explore ex vivo drug response testing without major surgical intervention [50, 51], although the success rate and time to establish cultures for comprehensive testing are still bottlenecks.

## Conclusions

In summary, our data strongly argue for including additional cell health parameters in drug sensitivity testing readouts for cell lines and primary cancer cells. By adding a simple cell death detection that is easily multiplexed with standard cell viability readouts one can detect a new level of heterogeneity of cellular responses. We expect that the combination of cell viability and cell death readouts will provide a more predictive measurement than cell viability alone. Our data provide further insights into the observations, where several targeted investigational drug classes, which have proven difficult to translate into effective and approved therapies, often show reversible cytostatic and antimetabolic effects rather than cancer cell-specific cytotoxicity in the in vitro model systems.

## Methods

### Cell lines

Human breast cancer cell lines used in this study were BT-474, BT-549, CAL-120, CAL-148, CAL-51, CAL-85-1, DU-4475, HCC-1143, HCC-1599, HCC-1937, HDQ-P1, Hs-578-T, MDA-MB-231, MDA-MD-436, MDA-MB-453, MDA-MB-468, MFM-223, MCF10A and SK-BR-3. The cell lines were obtained from DSMZ or ATCC collections and maintained at 37 °C with 5 % CO_2_ in a humidified incubator, according to provider’s instructions (Additional file 1: Table S1). The cell lines were grown in larger volume to make assay ready cells, tested for mycoplasma using PCR based test kit and frozen in several ampules. Each experiment was performed from unique assay ready cells (same passage). RPMI, DMEM and McCoy media were purchased from Lonza, Life Technologies and SigmaAldrich respectively.

### Drug Sensitivity and Resistance Testing

The DSRT platform used by Pemovska et al. [19] for screening leukemia cells was adapted for breast cancer cell lines. The chemicals used in this study are listed in Additional file 2: Table S2. The compounds were plated in 5 different concentrations in 10-fold dilutions covering a 10,000-fold concentration range centered around a compound-specific relevant cellular activity concentration (e.g. 1–10,000 nM for a compound with an ontarget cellular half-maximal effect of about 100 nM) on black clear bottom 384-well plates (Corning #3712) using an Echo 550 Liquid Handler (Labcyte). As negative and positive controls 0.1 % dimethyl sulfoxide (DMSO) and 100 μM benzethonium chloride were used, respectively. The pre-drugged plates were stored in pressurized Storage Pods (Roylan Developments Ltd.) filled with inert nitrogen gas. All subsequent liquid handling was performed using a MultiDrop Combi dispenser (Thermo Scientific). The pre-dispensed chemicals were dissolved in 5 μl of culture medium per well, with or without CellTox Green (1:2,000 final volume) depending on the experiment, for 1 h on an orbital shaker, and 20 μl cell suspension per well was seeded in the drugged plates, resulting in the final cell densities as listed in Additional file 1: Table S1. After 72 h incubation, cell viability and cytotoxicity were measured. When multiplexed, cell death was first assessed by measuring fluorescence (485/520 nm excitation/emission filters) signal from CellTox Green. Twenty-five μl of CellTiter-Glo (Promega) reagent was subsequently added per well, and luminescence was recorded using a PheraStar plate reader (BMG Labtech) after 10 min incubation at room temperature. When CellTiter-Blue was used as viability measurement, 2.5 μl of the reagent was added, incubated in 37 °C for 2 h and fluorescence (560/590 nm excitation/emission filters) signal was recorded. All the percent inhibition, EC50 and DSS values from each viability and cytotoxicity measurements are listed in Additional file 13: Table S6. Cell confluency was monitored and calculated using IncuCyte live cell microscopes (Essen Bioscience).

### Drug effect reversibility test

Cells were treated with compounds at concentrations 10X their growth inhibition IC_50_ for 72 h in duplicate, after which the compounds were washed away from one set, and replenished on the other set. The cells were cultured for 6 more days. Confluence of cells was monitored and recorded using an IncuCyte live cell microscope.

### Data analysis

The raw fluorescence intensity/luminescence data was analyzed using the Dotmatics Studies software. Each plate was first normalized against the positive and negative controls and the Z’-factors were then used to control the quality of each plate. Data was plotted as percent inhibition of viability and/or percent toxicity versus drug concentration yielding dose response curves. The values for EC_50_, slope and maximum asymptote were calculated for each drug (raw data are listed in Additional file 13: Table S6). The dose response data was further used to calculate the quantitative drug sensitivity scores (DSS) for each compound, as described previously [19, 20]. For distinguishing TNBC-selective responses from the broadly toxic effects, we calculated the differential score (selective DSS) by using the average DSS of a panel of 150 cell lines (consisting of seven different tissue types) as control for viability readouts and the average of DSS of 30 cell lines (three tissue types) for cytotoxicity readouts (Additional file 4: Table S4).

### Scoring and clustering of DSRT data

Unsupervised hierarchical clustering of the drug sensitivity profiles was performed with the Cluster 3.0 application (http://bonsai.hgc.jp/~mdehoon/software/cluster/) using complete-linkage clustering and Spearman rank and Euclidean distance measures of the drug and cell line profiles, respectively. Heat maps and dendrograms were visualized using Java TreeView (http://jtreeview.sourceforge.net/).

#### Synergy assessment

The Bliss independence model [52, 53] was used to define the pairwise drug combination effects in concentration combination matrices. The Bliss score was normalized in a way that values less than zero represent antagonism and values larger than zero represent synergism. The combination matrix plots were created with the Bliss score for each interaction of the two drugs in different concentrations. To validate our finding using another synergy model, we implemented recently developed Zero Interaction Potency (ZIP) model to score the drug combination effects (so-called delta score), which combines the advantages of both the Loewe and Bliss models in this type of combination testing setups [54]. Delta score analysis confirmed the findings from the Bliss score analysis (Additional file 14: Figure S7).

### Bioinformatic analyses

The processed values from Reverse Phase Protein Array (RPPA) intensity data for 70 (phospho) proteins with fully validated antibodies [15] were assessed using the t-test to explore whether there was a significant difference between the groups of responders and non-responders. The groups of cell lines were defined based on the average cytotoxic drug response (DSS) to mitotic or proteasome inhibitors. Proteins with significant difference between groups (p ≤ 0.05) are shown in Fig. 6b and Additional file 7: Figure S3. The mutation data for the cell lines used in this study were obtained from the COSMIC Cell Line Project database (http://cancer.sanger.ac.uk/cell_lines).

## Additional files

Additional file 1: Table S1. Information on cell lines used in the study. (XLSX 55 kb) Additional file 2: Table S2. List of compounds screened against cell lines in the study. (XLSX 47 kb) Additional file 3: Table S3. Average DSS calculated for the compounds screened using different viability and toxicity measure. (XLSX 53 kb) Additional file 4: Table S4. Average control DSS for the 301 compounds based on viability (average of 150 cell lines comprising 7 tissue types) and cytotoxicity (average of 30 cell lines comprising 3 tissue types) readouts. (XLSX 54 kb) Additional file 5: Figure S1. Full heat map showing the 16 TNBC cell lines clustering based on drug responses assessed via cytotoxicity (CellTox Green) readout. The area highlighted in green box is the representative heat map shown in Fig. 2. (PDF 98 kb) Additional file 6: Figure S2. Full heat map showing the 16 TNBC cell lines clustering based on drug responses assessed via viability (CellTiter-Glo) readout. (PDF 81 kb) Additional file 7: Figure S3. (A) Heat map highlighting/differentiating the proteasome inhibitors sensitive and insensitive cell lines based on cytotoxicity exhibited by two proteasome inhibitors. (B) Box plot showing the differential expression of protein and phosphoprotein levels (published data) between mitotic inhibitor sensitive and insensitive cell lines. (PDF 104 kb) Additional file 8: Figure S4. ATP-competitive mTOR inhibitors antagonize the effect of diverse classes of compounds in CAL-51. (A) Scatter plot of DSS scored for combinatory effect of different compounds along with dactolisib (Y-axis) on CAL-51 cell line compared to their single compound effect (X-axis). The right-hand plot represents the DSS computed using viability assay and the left plot represents cell death assay. Data points with DSS difference more than 10 are highlighted with different colors representing different classes of agents as listed in the color legend. (PDF 118 kb) Additional file 9: Figure S5. Cell line specific synergistic drug combination effects. Blue 8 × 8 blue matrices show % viability inhibition whereas red matrices show % cell death as similar represented in Fig. 7 and likewise 7 × 7 matrix represents synergy score. (A) Combining dactolisib and everolimus resulted in enhancing both viability inhibition and cytotoxicity in CAL-148 and DU4475 cell lines and only increased viability inhibition in MDA-MB-231 but did not show any additive effect in cytotoxicity. (B) The synergistic effect of combining trametinib with iniparib and ponatinib was only seen in DU4475 but not in CAL-148 cell line. (PDF 118 kb) Additional file 10: Table S5. Percent inhibition data of drug combination matrices in Fig. 7 & S5. (XLSX 64 kb) Additional file 11: Figure S8. Time dependent combination effect of dactolisib and trametinib in DU4475 and MDA-MB-231 cell lines. Each matrix represents toxicity based delta score combination plot (red being synergistic and green being antagonistic). The toxicity readout was measured after 96 h from onset of screen disregarding different subsequent combination of drugs in different times to monitor (A) both drugs added together at 0 h, (B) dactolisib added at the onset of experiment and then trametinib combined after 24 h, (C) dactolisib added at the onset of experiment and then trametinib combined after 48 h. (PDF 145 kb) Additional file 12: Figure S6. 72 h end point drug screening results highly correlate with the longer drug exposer assays. DSRT was performed in CAL-51 cells lines and the drugs effect was followed over time for 168 h. (A) Heat map showing the effects of drug after exposure for 72 h, 120 h and 168 h. (B) Scatter plot showing the correlation of DSS calculated for 72 h and 120 h incubation. (C) Scatter plot comparing 72 h and 168 h incubation. (PDF 115 kb) Additional file 13: Table S6. Raw screening data using all the viability and toxicity readouts. (XLSX 1970 kb) Additional file 14: Figure S7. Drug combination effect reanalyzed using delta score for the combination matrix data shown in Fig. 7 and Fig. S5. Values less than zero represent antagonism (green color) and greater than zero represent synergism (red color). The table in the right-bottom corner shows the average delta score for each drug combination matrix. (PDF 183 kb)

## Abbreviations

BL1: basal-like 1
BL2: basal-like 2
CDK: cyclin-dependent kinase
DMSO: dimethyl sulfoxide
DSRT: drug sensitivity and resistance testing
DSS: drug sensitivity score
ER: estrogen receptor
HSP90: heat shock protein 90
IM: immunomodulator
LAR: luminal androgen receptor positive
M: mesenchymal-like
MSL: mesenchymal stem cell-like
PARP: Poly-ADP-ribose polymerase
PR: progesterone receptor
RPPA: reverse phase protein array
sDSS: selective DSS
TNBC: triple negative breast cancer
UNC: unclassified

## References

[1] Kreike B (2007). Gene expression profiling and histopathological characterization of triple-negative/basal-like breast carcinomas. Breast Cancer Res.

[2] Pal SK, Childs BH, Pegram M (2011). Triple negative breast cancer: unmet medical needs. Breast Cancer Res Treat.

[3] Bauer KR (2007). Descriptive analysis of estrogen receptor (ER)-negative, progesterone receptor (PR)-negative, and HER2-negative invasive breast cancer, the so-called triple-negative phenotype: a population-based study from the California cancer Registry. Cancer.

[4] Lehmann BD (2011). Identification of human triple-negative breast cancer subtypes and preclinical models for selection of targeted therapies. J Clin Invest.

[5] Burstein MD (2015). Comprehensive genomic analysis identifies novel subtypes and targets of triple-negative breast cancer. Clin Cancer Res.

[6] Elsawaf Z (2013). Biological subtypes of triple-negative breast cancer are associated with distinct morphological changes and clinical behaviour. Breast.

[7] Lehmann BD, Pietenpol JA (2014). Identification and use of biomarkers in treatment strategies for triple-negative breast cancer subtypes. J Pathol.

[8] Mayer IA (2014). New strategies for triple-negative breast cancer--deciphering the heterogeneity. Clin Cancer Res.

[9] Cancer Genome Atlas, N (2012). Comprehensive molecular portraits of human breast tumours. Nature.

[10] Shah SP (2012). The clonal and mutational evolution spectrum of primary triplenegative breast cancers. Nature.

[11] Audeh MW (2014). Novel treatment strategies in triple-negative breast cancer: specific role of poly(adenosine diphosphate-ribose) polymerase inhibition. Pharmgenomics Pers Med.

[12] Barretina J (2012). The Cancer Cell Line Encyclopedia enables predictive modelling of anticancer drug sensitivity. Nature.

[13] Garnett MJ (2012). Systematic identification of genomic markers of drug sensitivity in cancer cells. Nature.

[14] Heiser LM (2012). Subtype and pathway specific responses to anticancer compounds in breast cancer. Proc Natl Acad Sci U S A.

[15] Daemen A (2013). Modeling precision treatment of breast cancer. Genome Biol.

[16] Lawrence RT (2015). The proteomic landscape of triple-negative breast cancer. Cell Rep.

[17] Muellner MK (2015). Targeting a cell state common to triple-negative breast cancers. Mol Syst Biol.

[18] Haibe-Kains B (2013). Inconsistency in large pharmacogenomic studies. Nature.

[19] Pemovska T (2013). Individualized systems medicine strategy to tailor treatments for patients with chemorefractory acute myeloid leukemia. Cancer Discov.

[20] Yadav B (2014). Quantitative scoring of differential drug sensitivity for individually optimized anticancer therapies. Sci Rep.

[21] Chiaraviglio L, Kirby JE (2014). Evaluation of impermeant, DNA-binding dye fluorescence as a real-time readout of eukaryotic cell toxicity in a high throughput screening format. Assay Drug Dev Technol.

[22] Barton VN (2015). Multiple molecular subtypes of triple-negative breast cancer critically rely on androgen receptor and respond to enzalutamide in vivo. Mol Cancer Ther.

[23] Duncan JS (2012). Dynamic reprogramming of the kinome in response to targeted MEK inhibition in triple-negative breast cancer. Cell.

[24] Carracedo A (2008). Inhibition of mTORC1 leads to MAPK pathway activation through a PI3K-dependent feedback loop in human cancer. J Clin Invest.

[25] Ocana A (2014). Activation of the PI3K/mTOR/AKT pathway and survival in solid tumors: systematic review and meta-analysis. PLoS One.

[26] Ali K (2014). Inactivation of PI(3)K p110delta breaks regulatory T-cell-mediated immune tolerance to cancer. Nature.

[27] Spencer A (2014). The novel AKT inhibitor afuresertib shows favorable safety, pharmacokinetics, and clinical activity in multiple myeloma. Blood.

[28] Reyland ME (2009). Protein kinase C isoforms: Multi-functional regulators of cell life and death. Front Biosci.

[29] Lee CW (2008). Molecular dependence of estrogen receptor-negative breast cancer on a notch-survivin signaling axis. Cancer Res.

[30] Yamaguchi N (2008). NOTCH3 signaling pathway plays crucial roles in the proliferation of ErbB2-negative human breast cancer cells. Cancer Res.

[31] Cheng KW (2004). The RAB25 small GTPase determines aggressiveness of ovarian and breast cancers. Nat Med.

[32] Real PJ (2002). Resistance to chemotherapy via Stat3-dependent overexpression of Bcl-2 in metastatic breast cancer cells. Oncogene.

[33] Tabuchi Y (2009). Resistance to paclitaxel therapy is related with Bcl-2 expression through an estrogen receptor mediated pathway in breast cancer. Int J Oncol.

[34] Barre B (2007). The STAT3 oncogene as a predictive marker of drug resistance. Trends Mol Med.

[35] Yu D (2001). Mechanisms of ErbB2-mediated paclitaxel resistance and trastuzumab-mediated paclitaxel sensitization in ErbB2-overexpressing breast cancers. Semin Oncol.

[36] Dai Z (2006). Prediction of anticancer drug potency from expression of genes involved in growth factor signaling. Pharm Res.

[37] Dees EC, Orlowski RZ (2006). Targeting the ubiquitin-proteasome pathway in breast cancer therapy. Future Oncol.

[38] Prechtl A (2000). Tumor-biological factors uPA and PAI-1 as stratification criteria of a multicenter adjuvant chemotherapy trial in node-negative breast cancer. Int J Biol Markers.

[39] Small GW (2003). Repression of mitogen-activated protein kinase (MAPK) phosphatase-1 by anthracyclines contributes to their antiapoptotic activation of p44/42MAPK. J Pharmacol Exp Ther.

[40] Bostner J (2007). Amplification of CCND1 and PAK1 as predictors of recurrence and tamoxifen resistance in postmenopausal breast cancer. Oncogene.

[41] Noel EE (2010). The association of CCND1 overexpression and cisplatin resistance in testicular germ cell tumors and other cancers. Am J Pathol.

[42] Shi YY, Small GW, Orlowski RZ (2006). Proteasome inhibitors induce a p38 mitogenactivated protein kinase (MAPK)-dependent anti-apoptotic program involving MAPK phosphatase-1 and Akt in models of breast cancer. Breast Cancer Res Treat.

[43] Small GW (2007). Mitogen-activated protein kinase phosphatase-1 is a mediator of breast cancer chemoresistance. Cancer Res.

[44] Steelman LS (2008). Suppression of PTEN function increases breast cancer chemotherapeutic drug resistance while conferring sensitivity to mTOR inhibitors. Oncogene.

[45] Fatehullah A, Tan SH, Barker N (2016). Organoids as an in vitro model of human development and disease. Nat Cell Biol.

[46] Campbell JJ (2011). A multifunctional 3D co-culture system for studies of mammary tissue morphogenesis and stem cell biology. PLoS One.

[47] Walsh AJ (2014). Quantitative optical imaging of primary tumor organoid metabolism predicts drug response in breast cancer. Cancer Res.

[48] Chapman S (2010). Human keratinocytes are efficiently immortalized by a Rho kinase inhibitor. J Clin Invest.

[49] Brown DD (2015). Developing in vitro models of human ductal carcinoma in situ from primary tissue explants. Breast Cancer Res Treat.

[50] Yu M (2014). Cancer therapy. Ex vivo culture of circulating breast tumor cells for individualized testing of drug susceptibility. Science.

[51] Crystal AS (2014). Patient-derived models of acquired resistance can identify effective drug combinations for cancer. Science.

[52] Bliss CI (1939). The toxicity of poisons applied jointly1. Ann Appl Biol.

[53] Zhao W (2014). A New Bliss Independence Model to Analyze Drug Combination Data. J Biomol Screen.

[54] Yadav B (2015). Searching for Drug Synergy in Complex Dose–response Landscapes Using an Interaction Potency Model. Comput Struct Biotechnol J.

