# Supplementary figures and images for: Identification of selective cytotoxic and synthetic lethal drug responses in triple negative breast cancer cells
Source: Mol Cancer. 2016 May 10;15:34. doi: 10.1186/s12943-016-0517-3 (PMC4862054; doi:10.1186/s12943-016-0517-3)

# Gautam\_Molecular Cancer\_Figure S1

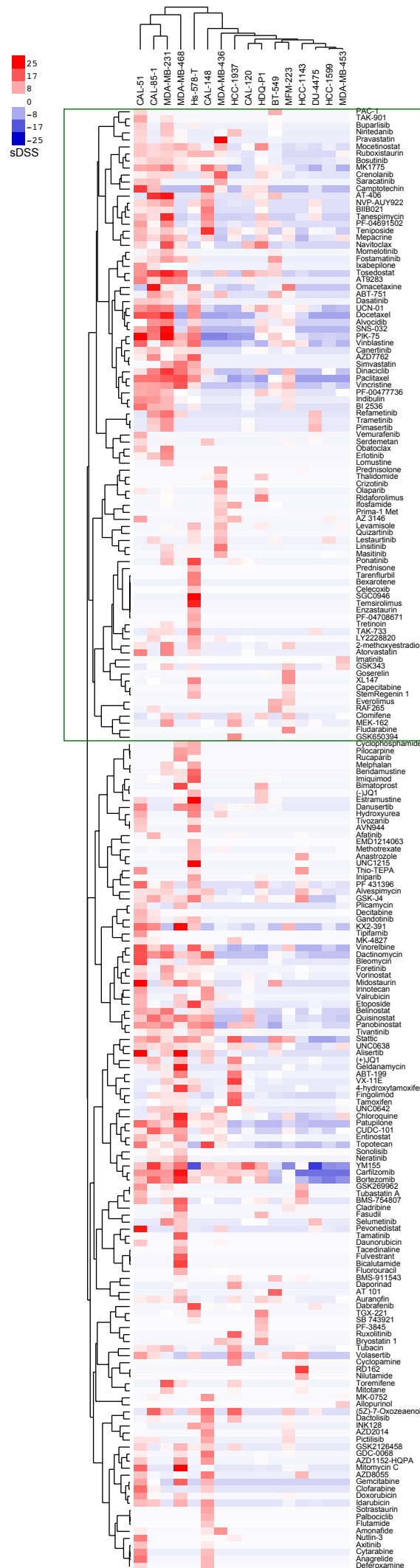

Supplement: Additional file 5: Figure S1. — Full heat map showing the 16 TNBC cell lines clustering based on drug responses assessed via cytotoxicity (CellTox Green) readout. The area highlighted in green box is the representative heat map shown in Fig. 2. (PDF 98 kb) [file 12943_2016_517_MOESM5_ESM.pdf]

Gautam\_Molecular Cancer\_Figure S2

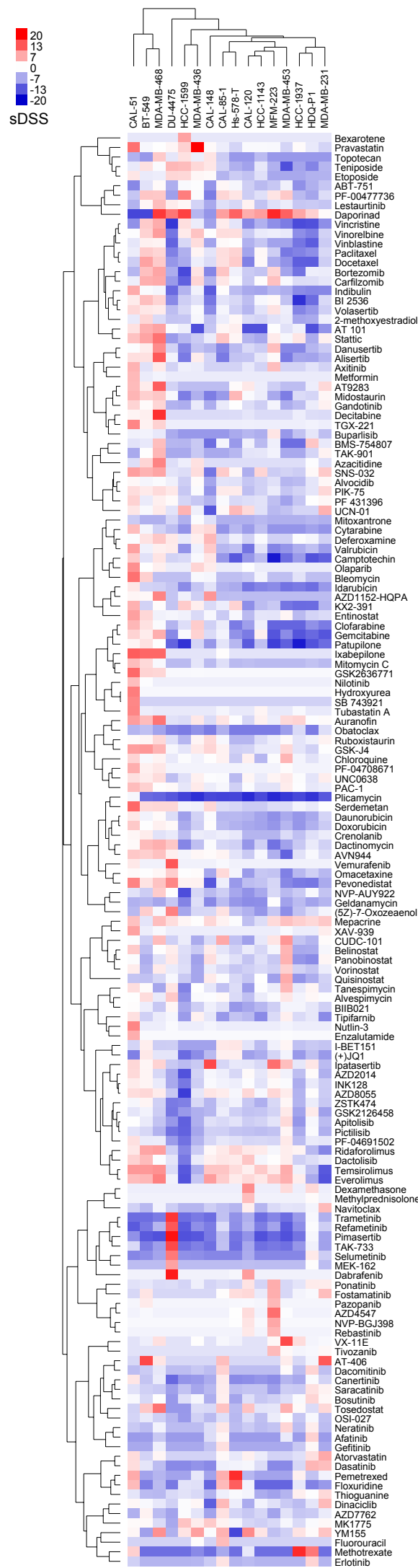

Supplement: Additional file 6: Figure S2. — Full heat map showing the 16 TNBC cell lines clustering based on drug responses assessed via viability (CellTiter-Glo) readout. (PDF 81 kb) [file 12943_2016_517_MOESM6_ESM.pdf]

## Gautam\_Molecular Cancer\_Figure S3

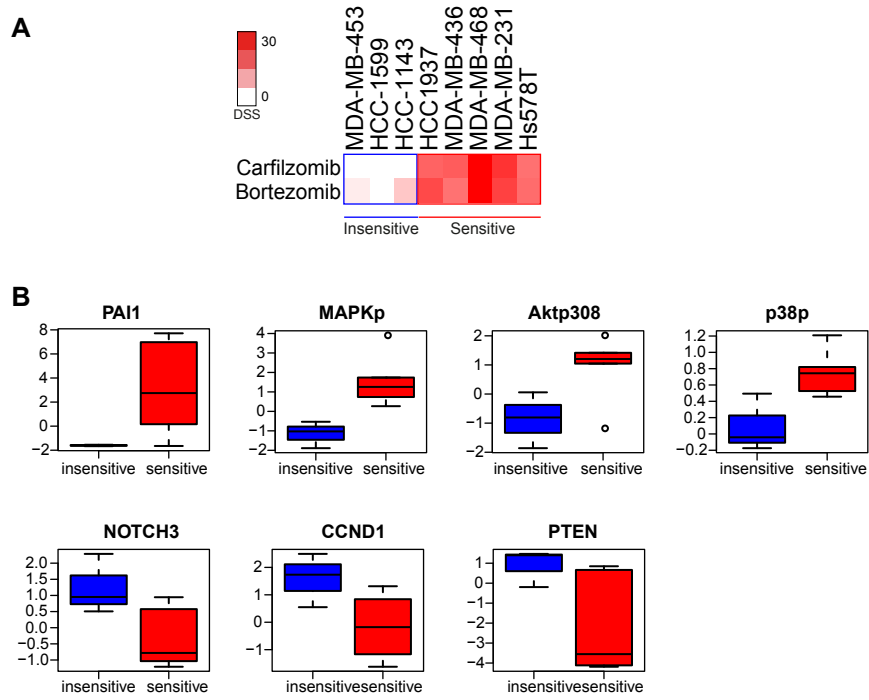

Supplement: Additional file 7: Figure S3. — (A) Heat map highlighting/differentiating the proteasome inhibitors sensitive and insensitive cell lines based on cytotoxicity exhibited by two proteasome inhibitors. (B) Box plot showing the differential expression of protein and phosphoprotein levels (published data) between mitotic inhibitor sensitive and insensitive cell lines. (PDF 104 kb) [file 12943_2016_517_MOESM7_ESM.pdf]

## Gautam Molecular Cancer Figure S5

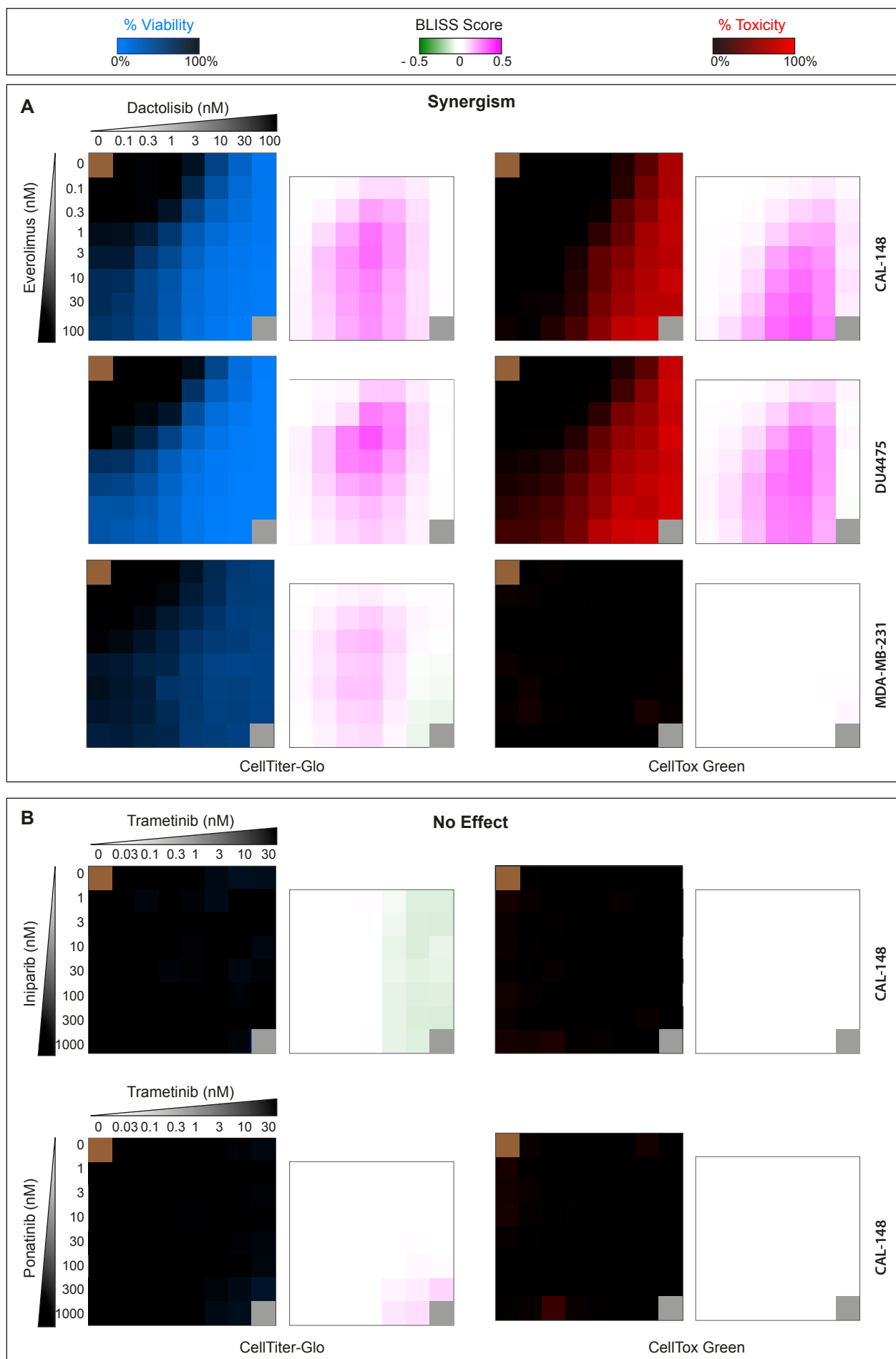

Supplement: Additional file 9: Figure S5. — Cell line specific synergistic drug combination effects. Blue 8 × 8 blue matrices show % viability inhibition whereas red matrices show % cell death as similar represented in Fig. 7 and likewise 7 × 7 matrix represents synergy score. (A) Combining dactolisib and everolimus resulted in enhancing both viability inhibition and cytotoxicity in CAL-148 and DU4475 cell lines and only increased viability inhibition in MDA-MB-231 but did not show any additive effect in cytotoxicity. (B) The synergistic effect of combining trametinib with iniparib and ponatinib was only seen in DU4475 but not in CAL-148 cell line. (PDF 118 kb) [file 12943_2016_517_MOESM9_ESM.pdf]

Gautam\_Molecular Cancer\_Figure S8

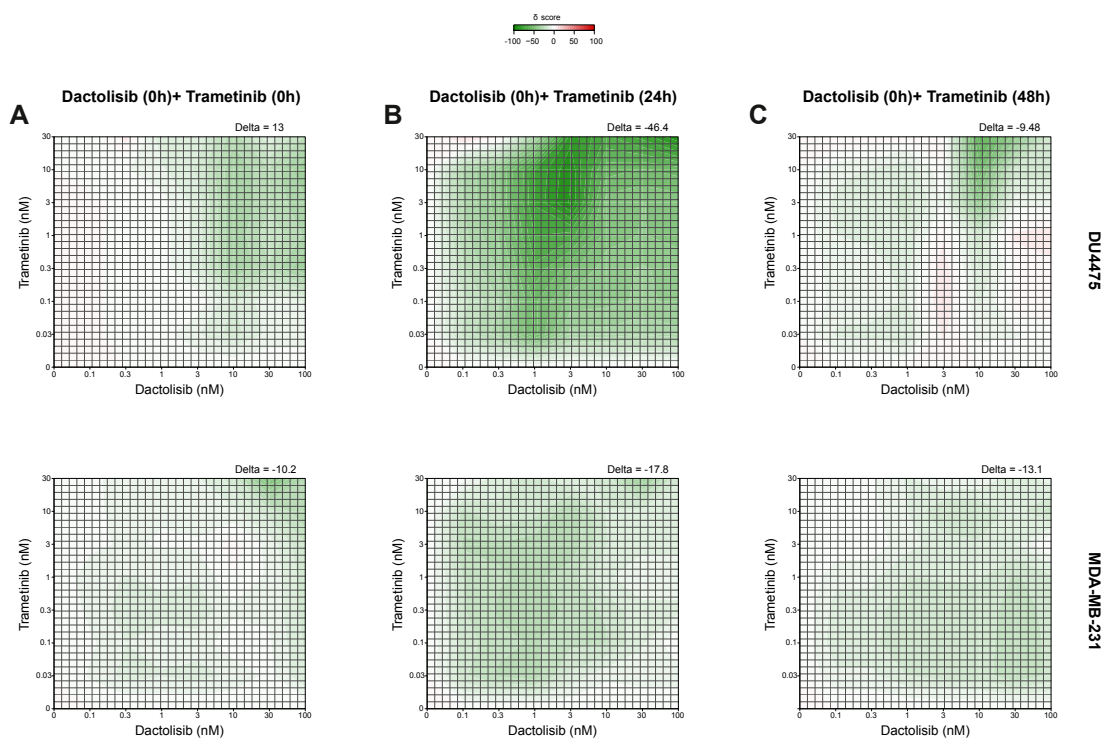

Supplement: Additional file 11: Figure S8. — Time dependent combination effect of dactolisib and trametinib in DU4475 and MDA-MB-231 cell lines. Each matrix represents toxicity based delta score combination plot (red being synergistic and green being antagonistic). The toxicity readout was measured after 96 h from onset of screen disregarding different subsequent combination of drugs in different times to monitor (A) both drugs added together at 0 h, (B) dactolisib added at the onset of experiment and then trametinib combined after 24 h, (C) dactolisib added at the onset of experiment and then trametinib combined after 48 h. (PDF 145 kb) [file 12943_2016_517_MOESM11_ESM.pdf]

## Gautam\_Molecular Cancer\_Figure S6

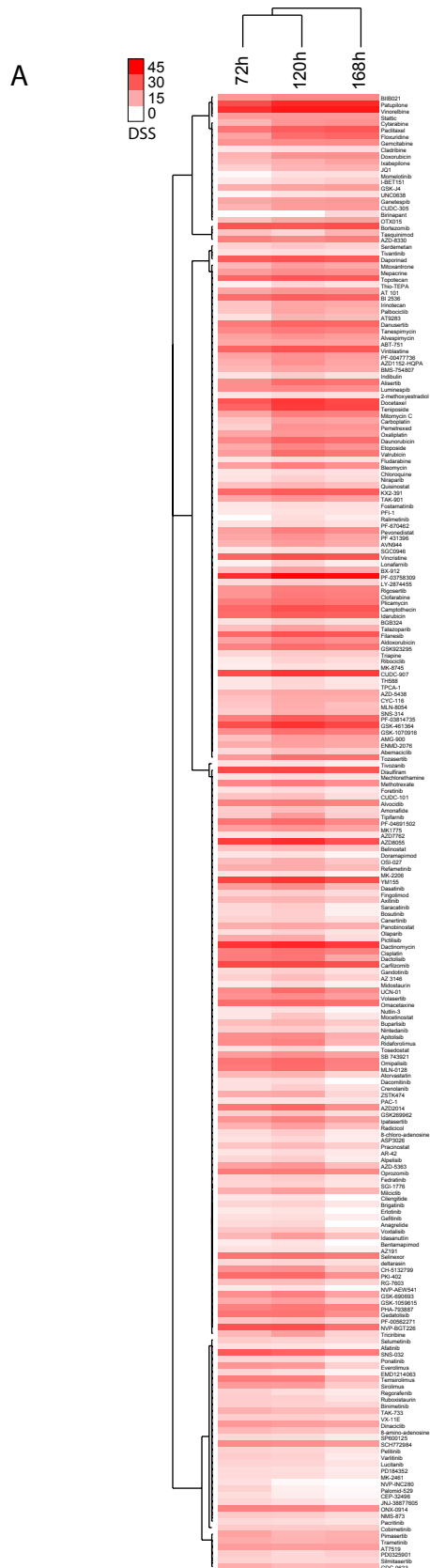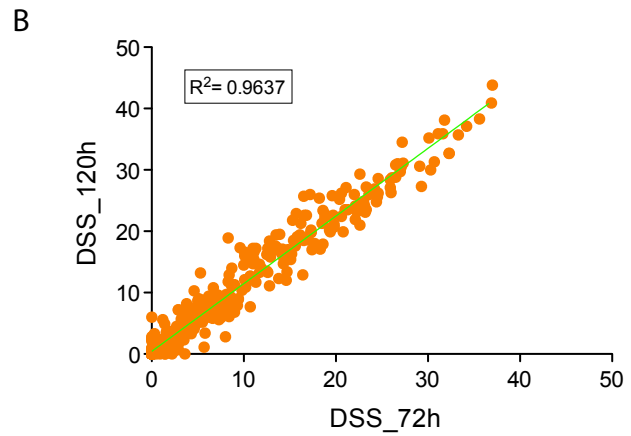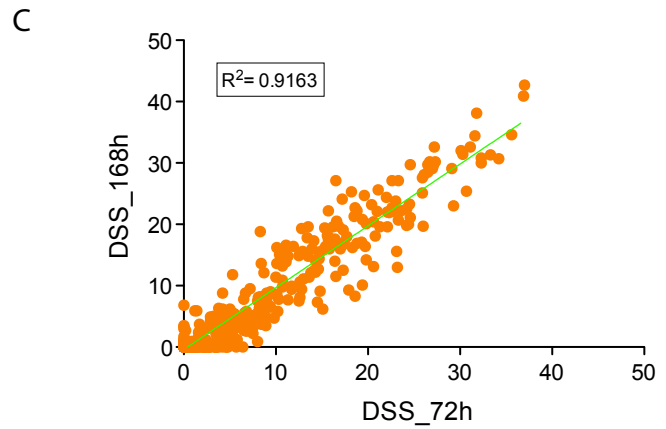

Supplement: Additional file 12: Figure S6. — 72 h end point drug screening results highly correlate with the longer drug exposer assays. DSRT was performed in CAL-51 cells lines and the drugs effect was followed over time for 168 h. (A) Heat map showing the effects of drug after exposure for 72 h, 120 h and 168 h. (B) Scatter plot showing the correlation of DSS calculated for 72 h and 120 h incubation. (C) Scatter plot comparing 72 h and 168 h incubation. (PDF 115 kb) [file 12943_2016_517_MOESM12_ESM.pdf]

# Gautam\_Molecular Cancer\_Figure S7

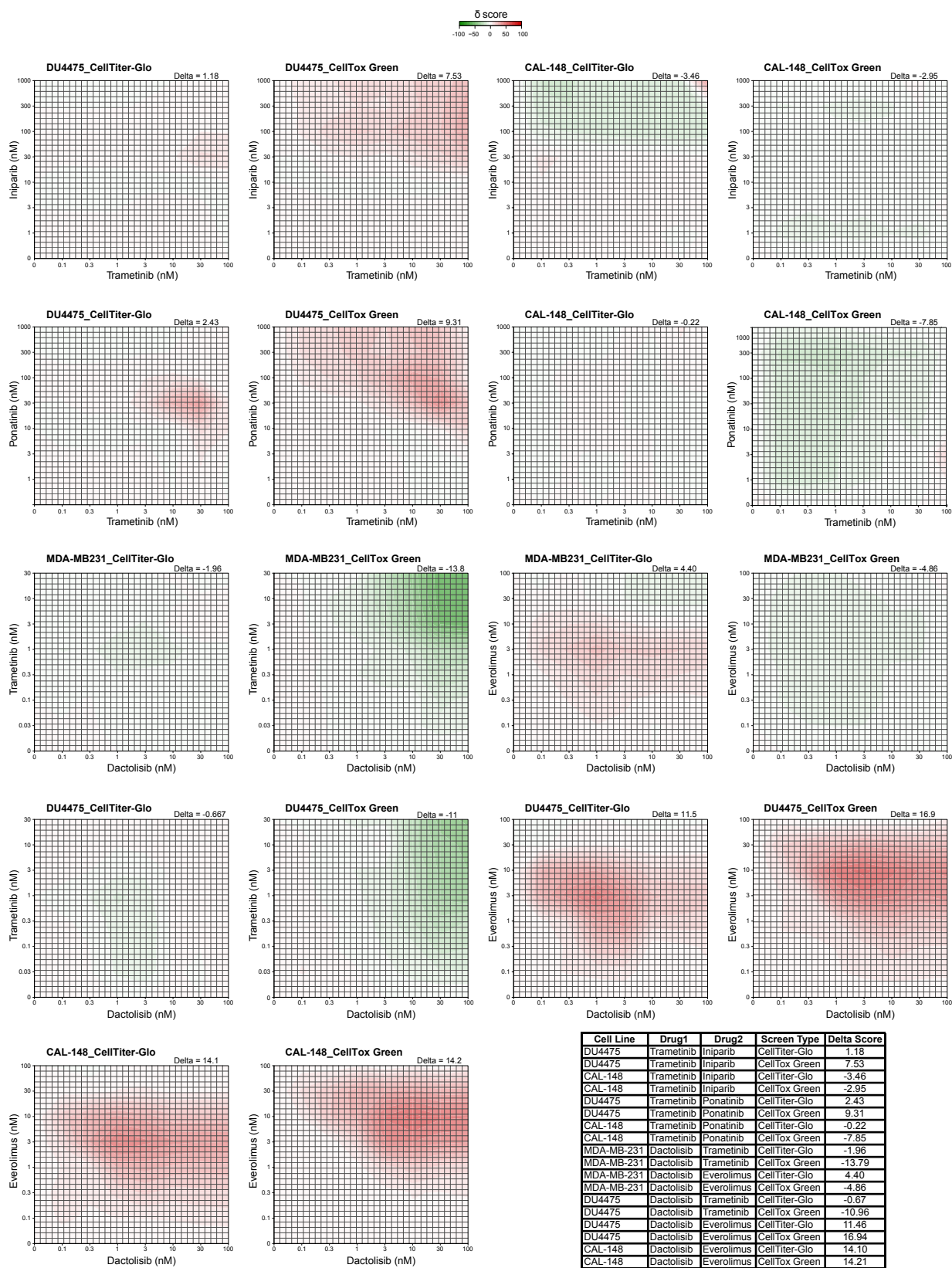

Supplement: Additional file 14: Figure S7. — Drug combination effect reanalyzed using delta score for the combination matrix data shown in Fig. 7 and Fig. S5. Values less than zero represent antagonism (green color) and greater than zero represent synergism (red color). The table in the right-bottom corner shows the average delta score for each drug combination matrix. (PDF 183 kb) [file 12943_2016_517_MOESM14_ESM.pdf]
