# Supplementary material for: Identification of selective cytotoxic and synthetic lethal drug responses in triple negative breast cancer cells
Source: Mol Cancer. 2016 May 10;15:34. doi: 10.1186/s12943-016-0517-3 (PMC4862054; doi:10.1186/s12943-016-0517-3)
Supplement: Additional file 8: Figure S4. — ATP-competitive mTOR inhibitors antagonize the effect of diverse classes of compounds in CAL-51. (A) Scatter plot of DSS scored for combinatory effect of different compounds along with dactolisib (Y-axis) on CAL-51 cell line compared to their single compound effect (X-axis). The right-hand plot represents the DSS computed using viability assay and the left plot represents cell death assay. Data points with DSS difference more than 10 are highlighted with different colors representing different classes of agents as listed in the color legend. (PDF 118 kb) [file 12943_2016_517_MOESM8_ESM.pdf]

Gautam_Molecular Cancer_Figure S4

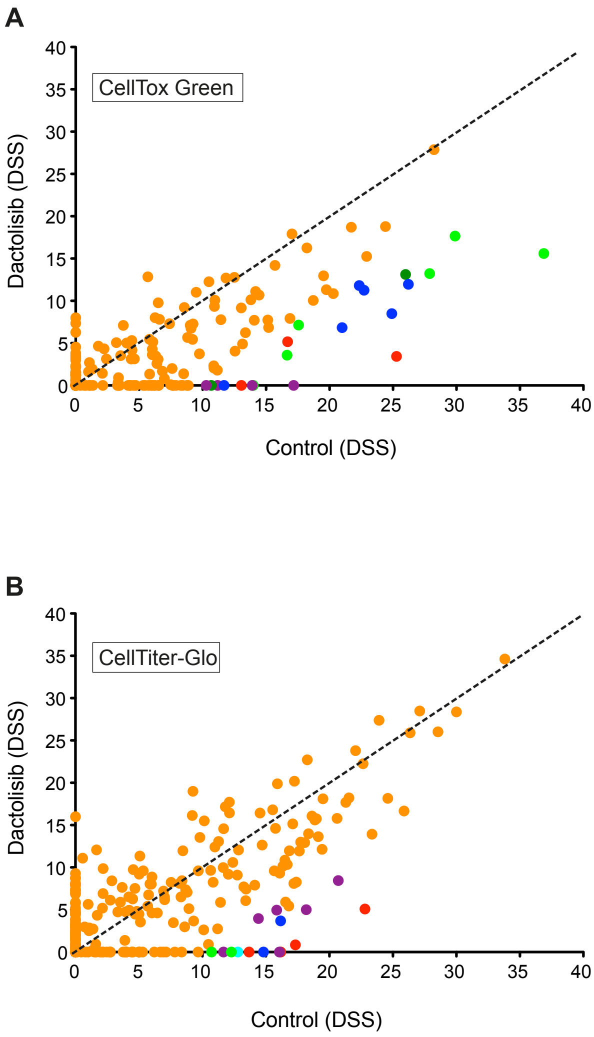

C

DRUG CLASSES	DRUG NAMES
Anti-metabolite	Cytarabine<br>Clofarabine<br>Pevonedistat
Anti-mitotic	Patupilone<br>Ixabepilone<br>Vinorelbin<br>Vinblastin<br>Paclitaxel<br>Docetaxel
Topoisomerase I inhibitor	Camptotechin<br>Irinotecan
Kinase inhibitor	Midostaurin<br>PF-431396<br>PIK-75<br>BI 2536<br>AT9283<br>Alisertib
Other inhibitors	Mytomycin C<br>Tipifarnib<br>Atorvastatin<br>Thio-TEPA
